# Isolated Hyponatremic Dehydration in the Setting of COVID-19–Associated Gastroenteritis in a Toddler

**DOI:** 10.1097/PG9.0000000000000002

**Published:** 2020-08-06

**Authors:** Mark Mahon, Penina Tarshish, Jeffrey Gershel, Noé D. Romo

**Affiliations:** From the Lewis M. Fraad Department of Pediatrics, Jacobi Medical Center, Albert Einstein College of Medicine.

Pediatric hallmarks of the current coronavirus disease 2019 (COVID-19) pandemic include a decreased incidence and less severe presentation when compared with adults, with the majority of reported pediatric cases classified as either mild or asymptomatic (1). Most pediatric cases reported symptoms of low-grade fever, dry nonproductive cough, and fatigue with some reporting nonpulmonary manifestations such as gastrointestinal (GI) symptoms (1–3).

Recently, there have been numerous reports from Europe and the United States of an emerging post–COVID-19 multisystem inflammatory syndrome in children (MIS-C) (4). There have also been a number of children with an acute COVID-19 infection presenting as gastroenteritis with nausea, vomiting, and diarrhea (2). The present patient is the first to be reported with isolated hyponatremic dehydration in the setting of COVID-19–associated gastroenteritis.

## CASE PRESENTATION

A 15-month-old male represented to the pediatric emergency department (ED) after a recent 2-day admission for gastroenteritis requiring intravenous rehydration. Three days after discharge, the patient reported continued intermittent episodes of nonbloody, nonbilous emesis associated with tactile fevers and nonbloody diarrhea for 4 consecutive days. The patient represented to the pediatric ED after reporting anuria for >24 hours.

**FIGURE 1.**
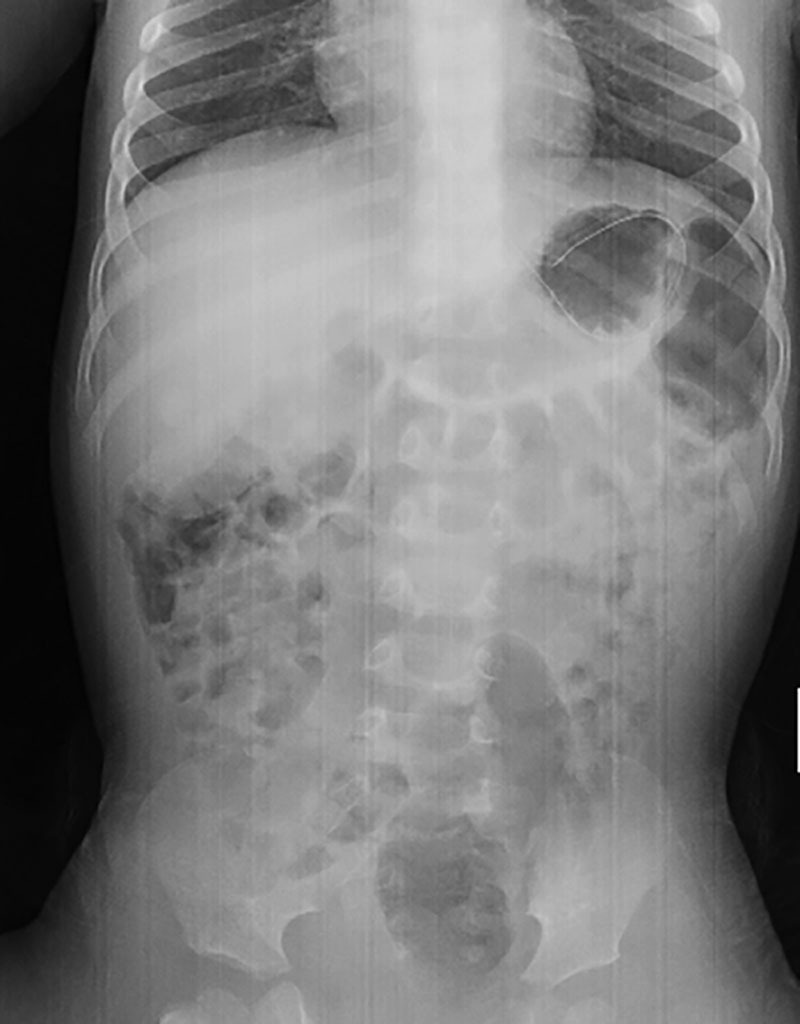
Plain abdominal radiograph with nonobstructive bowel gas pattern, demonstrating appropriate position of nasogastric tube.

On arrival to the ED, the patient was afebrile (98.6°F), tachycardic (129 bpm), in no respiratory distress with a respiratory rate of 28 and oxygen saturation 98%. Physical examination was notable for patients appearing fatigued, noninteractive, poor skin turgor, delayed capillary refill >2 seconds, with no associated rashes and normal abdominal examination. The patient was described as “not acting himself” and “more-sleepy” than usual. Laboratories were notable for white blood cell 7/nL, with lymphopenia (20%), hemoglobin/hematocrit of 12.1 g/dL and 36%, platelets 381/nL, sodium (Na) 124 mEq/L, chloride 76 mEq/L, anion gap (28 mEq/L) metabolic acidosis, blood urea nitrogen/creatinine of 14 mg/dL/0.4 mg/dL and a serum osmolality of 258 mOsm/L. Hepatic panel was normal. A nasogastric tube was placed with confirmation (Fig. 1), and the patient was given a 20 mL/kg normal saline bolus (320 mL total). As diarrhea persisted, our patient was admitted with the diagnosis of hyponatremic dehydration.

On the pediatrics floor, the patient was afebrile (98°F), tachycardic (123 bpm), normotensive (111/70 mm Hg), with a 1.7-kg weight loss noted since prior admission. Physical examination was unchanged. Further laboratory testing notable for phosphorus 3.7 mg/dL, magnesium 2 mg/dL, C-reactive protein 2.2 mg/L, urinary electrolytes with <20 mEq/L of Na and chloride. Reducing substances, occult blood and stool cultures were all negative. COVID-19 nasal swab polymerase chain reaction assay was sent as per protocol for all admissions and was positive.

**FIGURE 2.**
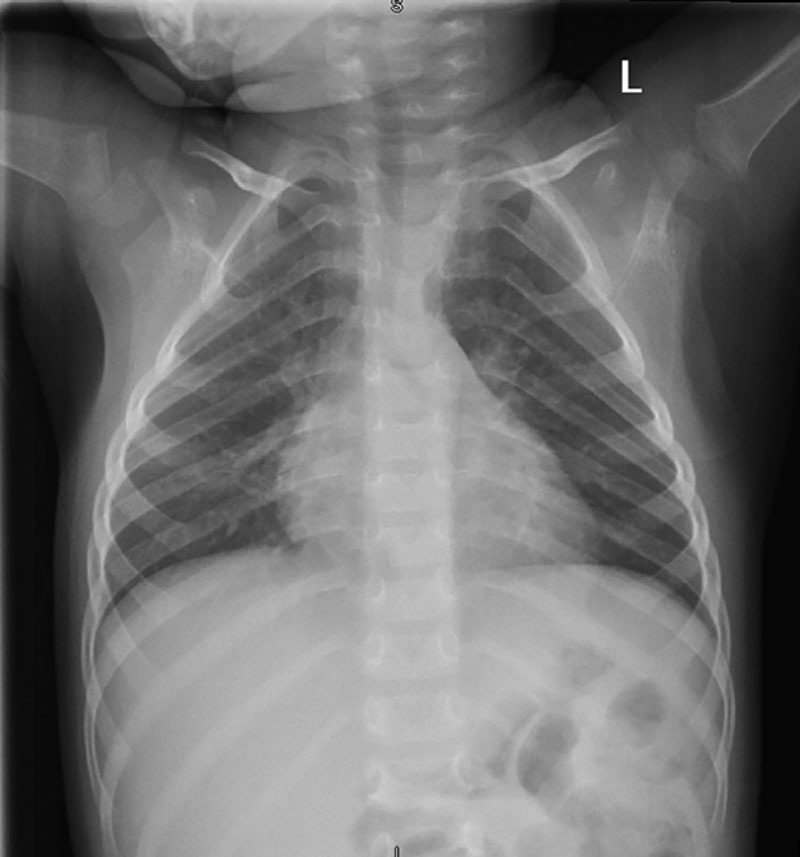
Plain anterior-posterior chest radiograph demonstrating bilateral perihilar interstitial markings.

The fluid deficit was estimated at 20% (1560 mL) and a Na deficit was calculated at 80 mEq. The patient was repleted over 24 hours with D5 normal saline 195 mL/hr for the first 8 hours, followed by 120 mL/hr over the latter 16 hours, which equated to a Na repletion rate of 5 and 2.5 mEq/h, respectively. A repeat basic metabolic panel 12 hours after admission showed a Na of 125 mEq/L and bicarbonate of 10.2 mEq/L which prompted the addition of 10 mEq/L of sodium bicarbonate to the solution. The patients’ Na normalized to 140 mEq/L by hospital day 3 and diarrhea resolved by hospital day 4. Throughout his hospitalization, the patient never developed any respiratory symptoms and had no oxygen requirement. A baseline chest radiograph (Fig. 2) demonstrated bilateral perihilar interstitial markers. By hospital day 4 (day 17 of illness), the patient was tolerating regular diet/fluids, at baseline mental status/activity and was discharged home. Three days after discharge, the patient had a televisit performed that reported an asymptomatic well-child.

## DISCUSSION

Until recent reports of MIS-C, symptoms of pediatric COVID-19 cases have been mild, with none as severe as seen in our patient (3). This patient presented early in the COVID-19 pandemic in New York City before MIS-C was defined, but in retrospect did not meet Centers for Disease Control and Prevention clinical criteria defined as age <21 years old with fever ≥100.4°F, laboratory evidence of inflammation with multisystem involvement, with no alternative diagnosis in the setting of confirmed severe acute respiratory syndrome coronavirus 2 (SARS-CoV-2) infection or known positive exposure within the previous 4 weeks. Our patient presented with isolated hyponatremic dehydration with COVID-19–associated gastroenteritis. The pathophysiology for COVID-19 GI illness may be related to the expression of angiotensin-converting enzyme 2 receptors. This known coronavirus entry binding site is expressed in both alveolar type 2 cells, ileal/colon enterocytes along with other organ systems (5). Recent evidence also suggests a 2-day delay between GI symptom onset and hospital admission compared with those with primary pulmonary symptoms (3). It is possible that limited clinical experience on disease manifestation resulted in decreased COVID-19 testing in patients with nonpulmonary manifestations such as GI symptoms, which may delay admission as was seen in our patient.

During the previous SARS-CoV outbreak (2003), which has 80% genomic similarity to COVID-19, watery diarrhea was present on admission in 20% of hospitalized cases, and 18% developed watery diarrhea while hospitalized (6,7). Postmortem analysis on 5 patients with GI manifestations in this cohort found normal gross examination and light microscopy, but identified viral particles consistent with coronavirus on electron microscopy in both small and large bowel biopsy specimens (6,7). These viral particles were confined to apical epithelial cells without evidence of villous atrophy to suggest a secondary cause of gastroenteritis (i.e., lactase deficiency) (6,7). The preservation of enterocyte structural integrity despite evidence of active SARS-CoV infection of the GI tract suggests the potential role of protein dysregulation or toxin production as the underlying etiology for GI manifestations, and not malabsorption or inflammation. It has also been shown that secondary alterations to gut microbiome composition can occur in response to immune dysregulation from extragastrointestinal illness such as respiratory disease (8). Another possible etiology for COVID-19–related hyponatremia may be the role of a syndrome of inappropriate antidiuretic hormone secretion (SIADH), resulting in water retention (9,10). SIADH has been a proposed etiology to explain why hyponatremia has been a presenting laboratory finding in 50% of adult COVID-19 patients (9). Hyponatremia as a predominant presenting laboratory finding is yet to be established in the pediatric COVID-19 literature. Our patient had a low serum osmolality and a urinary Na <20 mEq/L, which is not consistent with SIADH and suggests a hypovolemic hyponatremia from extrarenal losses to be the more likely underlying etiology.

This case raises another potential manifestation of COVID-19 infection in children, which may present as an isolated postinflammatory Na losing enteropathy, clinically differentiated from MIS-C. Our patient, with documented active COVID-19 infection, presented with isolated hyponatremic dehydration requiring fluid resuscitation and electrolyte repletion, despite normal inflammatory markers and no multisystem involvement. This case highlights the need for clinical awareness of nonpulmonary presentation(s) of COVID-19 in children, such as prolonged GI symptoms that may result in clinically significant electrolyte abnormalities.
